# The Netherlands as frontrunner of collaborative research in adult congenital heart disease

**DOI:** 10.1007/s12471-016-0893-8

**Published:** 2016-09-06

**Authors:** D. Robbers-Visser, B. J. M. Mulder

**Affiliations:** Department of Cardiology, Academic Medical Center, Amsterdam, The Netherlands

Adult congenital heart disease (ACHD) has become an important subspecialty in cardiology. Over the past 40 years, life expectancy of patients with congenital heart disease (CHD) has greatly increased, in particular by developments in the field of cardiac surgery together with improved perioperative management and care in outpatient clinics. Up until 6 decades ago, only 15 % of patients born with CHD survived into adulthood without surgical correction. Nowadays, over 90 % of these patients are expected to survive into adulthood [1]. Due to these developments, not only the population of adults with CHD is growing in numbers, but also the proportion of patients with complex CHD steadily increases. However, residual sequelae are frequent and re-interventions are often needed later in life. It has been estimated that approximately 50 % of the ACHD patients may face the prospect of future surgery, cardiac arrhythmias, heart failure and premature death [1]. Late morbidity may influence quality of life in a considerable proportion of this population and life-long cardiac follow-up is therefore required for nearly all CHD patients.

In Westernised countries, the need for specialised care for ACHD patients has long been recognised and the amount of ACHD centres in these countries outnumber the worldwide proposal of one ACHD centre per 10 million population [2]. In the Netherlands, tertiary referral centres collaborate with regional hospitals, creating a network of ACHD care as part of the CONCARE project [3].

In clinical care interdisciplinary collaboration between ACHD cardiologists, paediatric cardiologists, electrophysiologists, cardiac surgeons, specialised nurses, haematologists, psychologists and gynaecologists is of paramount importance. To aid clinical decision-making, both the American College of Cardiology/American Heart Association and the European Society of Cardiology (ESC) published guidelines on the management of grown-up congenital heart disease in 2008 and 2010 respectively [4, 5]. Not surprisingly, the level of evidence of the recommendations in these guidelines is only class C for almost all recommendations. Data for these recommendations are derived from observational and mostly retrospective studies. More data are needed to refine the current recommendations, resulting in level of evidence A or B recommendations. However, research is hampered by the heterogeneity of the ACHD patient population, resulting in small patient numbers per diagnosis, and a low mortality rate. Adequately powered, prospectively randomised trials and surrogate markers with good predictive value for mortality are needed. This requires collaboration in research, both at a national and international level.

**Fig. 1:**
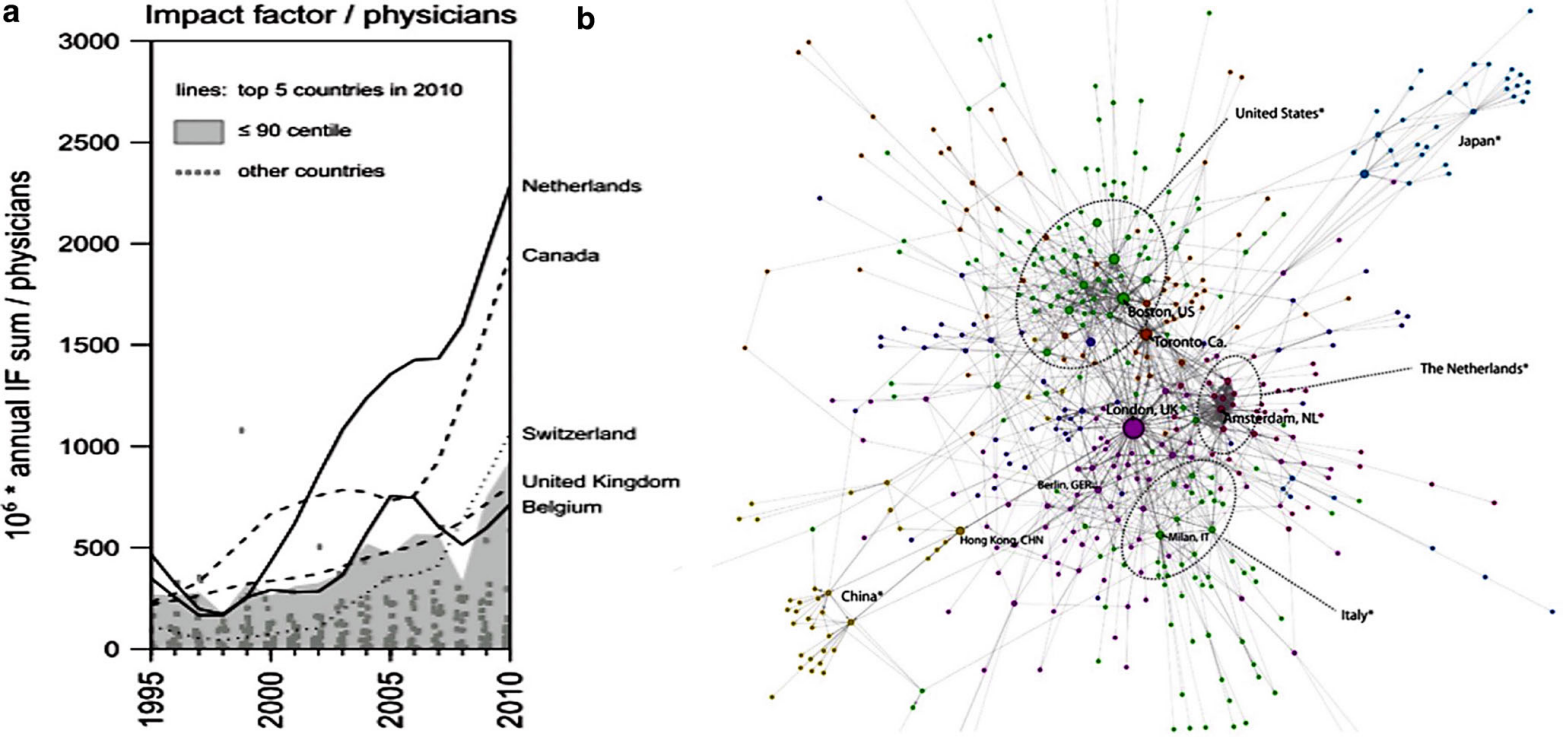
**a** Relation between national annual impact factor and the total amount of physicians. It shows that the Netherlands, Canada, Switzerland, the United Kingdom, and Belgium use their resources most efficiently to achieve high scientific output [2]. **b** Network graph of the city based data map. The size of the nodes corresponds to the degree of centrality of the node, the weight assigned to the ties corresponds to the cumulative impact factor of the particular link [7]

In a worldwide survey on the research output of ACHD centres, Kempny et al. demonstrated that the summed impact factor for ACHD publications has increased dramatically between 1995 and 2011 [2]. The Dutch ACHD centres yield a disproportionally large academic contribution given the demographic and economic characteristics of our country (Fig. 1a). The authors considered the remarkable increase of scientific output in the Netherlands to be due to the initiation of the nationwide registry on ACHD: CONCOR (CONgenital CORvitia). The CONCOR database was set up by the Netherlands Heart Institute (formerly ICIN) in 2001 in order to facilitate research on long-term outcome and genetic underlying causes [6]. At present, more than 16,000 adult patients have been included from around 100 participating hospitals. Numerous national and international studies have emerged from this registry, and CONCOR has become a global brand for collaborative research. This was acknowledged by Orwat et al. [7], who studied network analysis in ACHD research and showed that research collaboration is common, but unevenly distributed worldwide. In the Netherlands, Norway and Austria, more than 60 % of publications originate from collaborative research efforts, with by far the most publications from the Netherlands (145, 18 and 10 respectively). This compares with 38 % of the studies from the United States and United Kingdom. Fig. 1b shows a worldwide network graph of the city based data map, based on multicentre scientific output. In this way multiple international studies initiated by the Netherlands have been used to support the recommendations in the international ACHD guidelines. The emphasis has to shift to prospectively gathered data to generate more evidence-based guidelines (level A or B).

With the increasing implementation of electronic patient files and electronic healthcare data, more clinical information will become available for research purposes. How to deal with these Big Data is still under investigation, but this can be useful in predictive analytics and as an adjunct to the current registry data.

In this issue of the Netherlands Heart Journal, the variety of Dutch research in ACHD is clearly demonstrated. Continuous efforts for collaboration on a national and international level are needed to increase patient numbers and outcome measures and will provide important data to improve international guidelines and the care for ACHD patients.
